# Supplementary material for: Feeding Preference of Crapemyrtle Bark Scale (Acanthococcus lagerstroemiae) on Different Species
Source: Insects. 2020 Jun 28;11(7):399. doi: 10.3390/insects11070399 (PMC7412028; doi:10.3390/insects11070399)
Supplement: Supplementary file 1 [file insects-11-00399-s001.zip › Supplementary files_CMBS feeding preference 8 genera.docx]

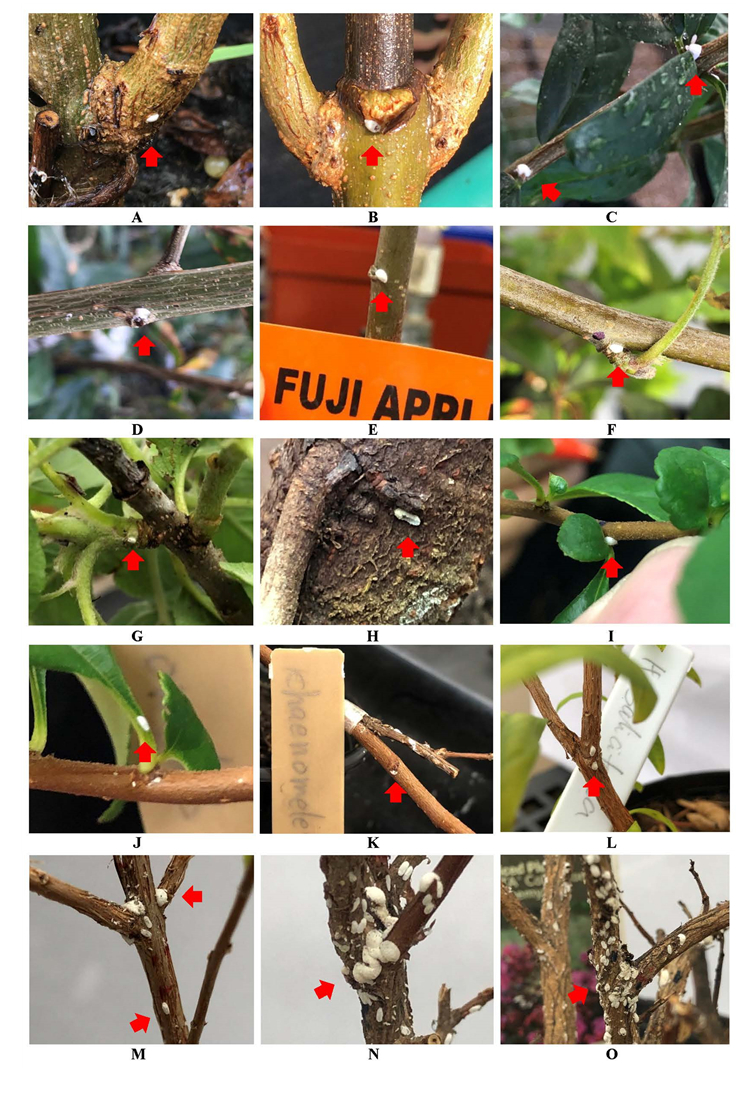


**Figure S1.** Infestation of *Acanthococcus* *lagerstromiae* (red arrows) found on *Punica* ‘Angel Red’ (A), *P.* ‘Sumbar’ (B), *P.* ‘Mollar’ (C), *P.* ‘Kandahar’ (D), *Malus domestica* ‘Fuji’ (E-F), *M. domestica* ‘Red Delicious’ (G), *M. angustifolia* (H), *Chaenomeles speciosa* (I-K), *Heimia salicifolia* (L-M), and *Lagerstroemia* ‘Spiced Plum’ (N-O) recorded from feeding preference studies in 2016 and 2019.

Table S1. The number of male pupae of *Acanthococcus lagerstromiae* on fourteen plant species and cultivars by time from June to December 2019.

| **Level** | **Least Sq Mean** |
| --- | --- |
| *Lagerstroemia* 'Spiced Plum',Week 20 | 634.33a ^Z^ |
| *Lagerstroemia* 'Spiced Plum',Week 18 | 579ab |
| *Lagerstroemia* 'Spiced Plum',Week 16 | 532.33abc |
| *Lagerstroemia* 'Spiced Plum',Week 22 | 497.33abc |
| *Lagerstroemia* 'Spiced Plum',Week 14 | 435.33bc |
| *Lagerstroemia* 'Spiced Plum',Week 12 | 424.67bc |
| *Lagerstroemia* 'Spiced Plum',Week 30 | 382.33c |
| *Lagerstroemia* 'Spiced Plum',Week 26 | 378.33c |
| *Lagerstroemia* 'Spiced Plum',Week 10 | 197.67d |
| *Lagerstroemia* 'Spiced Plum',Week 8 | 70.33de |
| *Heimia salicifolia*,Week 16 | 28.67de |
| *Lagerstroemia* 'Spiced Plum',Week 6 | 28.67de |
| *Heimia salicifolia*,Week 18 | 22.67de |
| *Heimia salicifolia*,Week 12 | 20de |
| *Heimia salicifolia*,Week 14 | 17.33de |
| *Heimia salicifolia*,Week 10 | 17.33de |
| *Lagerstroemia* 'Spiced Plum',Week 4 | 16.67e |
| *Heimia salicifolia*,Week 22 | 14de |
| *Heimia salicifolia*,Week 26 | 12.67de |
| *Heimia salicifolia*,Week 20 | 12.67de |
| *Heimia salicifolia*,Week 30 | 7.67de |
| *Malus domestica* 'Red Delicious',Week 20 | 5.33de |
| *Malus domestica* 'Red Delicious',Week 14 | 4.67de |
| *Malus domestica* 'Red Delicious',Week 18 | 4.67de |
| *Malus domestica* 'Fuji',Week 14 | 4de |
| *Heimia salicifolia*,Week 8 | 3.67de |
| *Malus domestica* 'Red Delicious',Week 12 | 3.67de |
| *Malus domestica* 'Fuji',Week 16 | 3.67de |
| *Malus domestica* 'Fuji',Week 18 | 3.67de |
| *Malus domestica* 'Red Delicious',Week 16 | 3.67de |
| *Malus domestica* 'Red Delicious',Week 22 | 3.67de |
| *Chaenomeles speciosa*,Week 18 | 3.33de |
| *Lagerstroemia* 'Spiced Plum',Week 3 | 3e |
| *Heimia salicifolia*,Week 6 | 3de |
| *Malus domestica* 'Red Delicious',Week 10 | 3de |
| *Heimia salicifolia*,Week 4 | 3de |
| *Chaenomeles speciosa*,Week 16 | 3de |
| *Malus domestica* 'Fuji',Week 12 | 3de |
| *Chaenomeles speciosa*,Week 22 | 2.67de |
| *Malus domestica* 'Fuji',Week 26 | 2.33de |
| *Chaenomeles speciosa*,Week 20 | 2de |
| *Malus domestica* 'Fuji',Week 10 | 1.67de |
| *Malus domestica* 'Red Delicious',Week 26 | 1.67de |
| *Malus domestica* 'Fuji',Week 20 | 1.67de |
| *Heimia salicifolia*,Week 3 | 1.33de |
| *Chaenomeles speciosa*,Week 26 | 1.33de |
| *Malus domestica* 'Fuji',Week 22 | 1.33de |
| *Malus domestica* 'Fuji',Week 30 | 1de |
| *Malus domestica* 'Red Delicious',Week 30 | 1de |
| *Chaenomeles speciosa*,Week 14 | 1de |
| *Malus angustifolia*,Week 14 | 1de |
| *Malus angustifolia*,Week 10 | 0.67de |
| *Malus domestica* 'Fuji',Week 4 | 0.67de |
| *Chaenomeles speciosa*,Week 30 | 0.67de |
| *Malus domestica* 'Fuji',Week 6 | 0.33de |
| *Malus angustifolia*,Week 8 | 0.33de |
| *Malus domestica* 'Fuji',Week 3 | 0.33de |
| *Malus domestica* 'Fuji',Week 8 | 0.33de |
| *Chaenomeles speciosa*,Week 12 | 0.33de |
| *Diospyros rhombifolia*,Week 3 | 0.33de |
| *Malus angustifolia*,Week 12 | 0.33de |
| *Malus angustifolia*,Week 30 | 0de |
| *Rubus* 'Navaho',Week 6 | 0de |
| *Rubus* 'Navaho',Week 20 | 0de |
| *Rubus* 'Navaho',Week 4 | 0de |
| *Rubus* 'Navaho',Week 3 | 0de |
| *Diospyros virginiana*,Week 30 | 0de |
| *Malus domestica* 'Red Delicious',Week 6 | 0de |
| *Rubus*’Arapaho’,Week 6 | 0de |
| *Malus angustifolia*,Week 6 | 0de |
| *Rubus fruticosus* ‘Prime Ark Freedom’,Week 6 | 0de |
| *Rubus idaeus* 'Dorman Red',Week 6 | 0de |
| *Rubus* 'Navaho',Week 26 | 0de |
| *Rubus* 'Navaho',Week 14 | 0de |
| *Rubus* 'Navaho',Week 12 | 0de |
| *Rubus*’Arapaho’,Week 4 | 0de |
| *Rubus idaeus* 'Dorman Red',Week 10 | 0de |
| *Diospyros rhombifolia*,Week 30 | 0de |
| *Rubus fruticosus* ‘Prime Ark Freedom’,Week 30 | 0de |
| *Rubus idaeus* 'Dorman Red',Week 30 | 0de |
| *Malus domestica* 'Red Delicious',Week 8 | 0de |
| *Rubus* 'Navaho',Week 10 | 0de |
| *Buxus harlandii*,Week 22 | 0de |
| *Buxus harlandii*,Week 20 | 0de |
| *Buxus microphylla* var. koreana x *Buxus sempervirens* ‘Green Gem’,Week 14 | 0de |
| *Rubus*’Arapaho’,Week 8 | 0de |
| *Rubus*’Arapaho’,Week 10 | 0de |
| *Buxus microphylla* var. koreana x *Buxus sempervirens* ‘Green Gem’,Week 16 | 0de |
| *Malus domestica* 'Red Delicious',Week 4 | 0de |
| *Rubus idaeus* 'Dorman Red',Week 8 | 0de |
| *Rubus*’Arapaho’,Week 3 | 0de |
| *Malus domestica* 'Red Delicious',Week 3 | 0de |
| *Rubus fruticosus* ‘Prime Ark Freedom’,Week 8 | 0de |
| *Rubus*’Arapaho’,Week 30 | 0de |
| *Rubus fruticosus* ‘Prime Ark Freedom’,Week 10 | 0de |
| *Rubus idaeus* 'Dorman Red',Week 3 | 0de |
| *Rubus idaeus* 'Dorman Red',Week 4 | 0de |
| *Buxus harlandii*,Week 26 | 0de |
| *Buxus microphylla* var. koreana x *Buxus sempervirens* ‘Green Gem’,Week 22 | 0de |
| *Diospyros virginiana*,Week 14 | 0de |
| *Buxus harlandii*,Week 12 | 0de |
| *Buxus harlandii*,Week 16 | 0de |
| *Diospyros virginiana*,Week 6 | 0de |
| *Rubus fruticosus* ‘Prime Ark Freedom’,Week 3 | 0de |
| *Rubus fruticosus* ‘Prime Ark Freedom’,Week 4 | 0de |
| *Buxus harlandii*,Week 14 | 0de |
| *Buxus microphylla* var. koreana x *Buxus sempervirens* ‘Green Gem’,Week 12 | 0de |
| *Malus angustifolia*,Week 4 | 0de |
| *Buxus harlandii*,Week 18 | 0de |
| *Buxus microphylla* var. koreana x *Buxus sempervirens* ‘Green Gem’,Week 26 | 0de |
| *Buxus microphylla* var. koreana x *Buxus sempervirens* ‘Green Gem’,Week 18 | 0de |
| *Diospyros virginiana*,Week 8 | 0de |
| *Malus angustifolia*,Week 3 | 0de |
| *Buxus microphylla* var. koreana x *Buxus sempervirens* ‘Green Gem’,Week 20 | 0de |
| *Chaenomeles speciosa*,Week 8 | 0de |
| *Diospyros rhombifolia*,Week 6 | 0de |
| *Diospyros rhombifolia*,Week 8 | 0de |
| *Buxus microphylla* var. koreana x *Buxus sempervirens* ‘Green Gem’,Week 10 | 0de |
| *Diospyros rhombifolia*,Week 14 | 0de |
| *Chaenomeles speciosa*,Week 10 | 0de |
| *Buxus harlandii*,Week 10 | 0de |
| *Diospyros virginiana*,Week 10 | 0de |
| *Chaenomeles speciosa*,Week 6 | 0de |
| *Rubus* 'Navaho',Week 22 | 0de |
| *Diospyros rhombifolia*,Week 10 | 0de |
| *Buxus harlandii*,Week 6 | 0de |
| *Buxus harlandii*,Week 8 | 0de |
| *Buxus microphylla* var. koreana x *Buxus sempervirens* ‘Green Gem’,Week 8 | 0de |
| *Diospyros rhombifolia*,Week 16 | 0de |
| *Diospyros rhombifolia*,Week 4 | 0de |
| *Diospyros virginiana*,Week 16 | 0de |
| *Diospyros rhombifolia*,Week 26 | 0de |
| *Diospyros virginiana*,Week 26 | 0de |
| *Rubus idaeus* 'Dorman Red',Week 14 | 0de |
| *Rubus*’Arapaho’,Week 14 | 0de |
| *Buxus microphylla* var. koreana x *Buxus sempervirens* ‘Green Gem’,Week 30 | 0de |
| *Diospyros rhombifolia*,Week 18 | 0de |
| *Malus angustifolia*,Week 26 | 0de |
| *Diospyros rhombifolia*,Week 12 | 0de |
| *Buxus microphylla* var. koreana x *Buxus sempervirens* ‘Green Gem’,Week 6 | 0de |
| *Rubus fruticosus* ‘Prime Ark Freedom’,Week 14 | 0de |
| *Rubus fruticosus* ‘Prime Ark Freedom’,Week 18 | 0de |
| *Buxus microphylla* var. koreana x *Buxus sempervirens* ‘Green Gem’,Week 3 | 0de |
| *Chaenomeles speciosa*,Week 3 | 0de |
| *Diospyros virginiana*,Week 12 | 0de |
| *Diospyros virginiana*,Week 18 | 0de |
| *Rubus idaeus* 'Dorman Red',Week 12 | 0de |
| *Buxus harlandii*,Week 4 | 0de |
| *Chaenomeles speciosa*,Week 4 | 0de |
| *Diospyros virginiana*,Week 3 | 0de |
| *Diospyros virginiana*,Week 4 | 0de |
| *Rubus*’Arapaho’,Week 12 | 0de |
| *Rubus fruticosus* ‘Prime Ark Freedom’,Week 12 | 0de |
| *Diospyros rhombifolia*,Week 22 | 0de |
| *Diospyros virginiana*,Week 22 | 0de |
| *Malus angustifolia*,Week 18 | 0de |
| *Buxus harlandii*,Week 3 | 0de |
| *Buxus microphylla* var. koreana x *Buxus sempervirens* ‘Green Gem’,Week 4 | 0de |
| *Rubus idaeus* 'Dorman Red',Week 18 | 0de |
| *Rubus*’Arapaho’,Week 18 | 0de |
| *Rubus fruticosus* ‘Prime Ark Freedom’,Week 26 | 0de |
| *Diospyros virginiana*,Week 20 | 0de |
| *Malus angustifolia*,Week 16 | 0de |
| *Rubus idaeus* 'Dorman Red',Week 26 | 0de |
| *Rubus fruticosus* ‘Prime Ark Freedom’,Week 16 | 0de |
| *Rubus idaeus* 'Dorman Red',Week 16 | 0de |
| *Rubus*’Arapaho’,Week 16 | 0de |
| *Rubus* 'Navaho',Week 18 | 0de |
| *Rubus*’Arapaho’,Week 26 | 0de |
| *Buxus harlandii*,Week 30 | 0de |
| *Diospyros rhombifolia*,Week 20 | 0de |
| *Rubus fruticosus* ‘Prime Ark Freedom’,Week 22 | 0de |
| *Rubus idaeus* 'Dorman Red',Week 22 | 0de |
| *Malus angustifolia*,Week 22 | 0de |
| *Rubus*’Arapaho’,Week 22 | 0de |
| *Rubus* 'Navaho',Week 16 | 0de |
| *Rubus* 'Navaho',Week 30 | 0de |
| *Rubus fruticosus* ‘Prime Ark Freedom’,Week 20 | 0de |
| *Malus angustifolia*,Week 20 | 0de |
| *Rubus*’Arapaho’,Week 20 | 0de |
| *Rubus* 'Navaho',Week 8 | 0de |
| *Rubus idaeus* 'Dorman Red',Week 20 | 0de |

^Z^ Means within each column followed by the same letter are not significantly different according to All Pairs, Tukey Honestly Significant Difference at 0.05 confidence level.

Table S2. The number of gravid females of *Acanthococcus lagerstromiae* on fourteen plant species and cultivars by time from June to December 2019.

| **Plant speices by time** | **Least Sq Mean** |
| --- | --- |
| *Lagerstroemia* 'Spiced Plum',Week 18 | 167a ^Z^ |
| *Lagerstroemia* 'Spiced Plum',Week 16 | 166a |
| *Lagerstroemia* 'Spiced Plum',Week 20 | 122b |
| *Lagerstroemia* 'Spiced Plum',Week 22 | 112.33b |
| *Lagerstroemia* 'Spiced Plum',Week 14 | 106.33b |
| *Lagerstroemia* 'Spiced Plum',Week 12 | 100.33b |
| *Lagerstroemia* 'Spiced Plum',Week 30 | 96.67b |
| *Lagerstroemia* 'Spiced Plum',Week 26 | 95.33b |
| *Lagerstroemia* 'Spiced Plum',Week 10 | 84.33bc |
| *Lagerstroemia* 'Spiced Plum',Week 8 | 56cd |
| *Lagerstroemia* 'Spiced Plum',Week 6 | 30.33de |
| *Heimia salicifolia*,Week 18 | 10de |
| *Heimia salicifolia*,Week 14 | 9.67de |
| *Heimia salicifolia*,Week 16 | 8.67ed |
| *Heimia salicifolia*,Week 22 | 8.33ed |
| *Heimia salicifolia*,Week 20 | 8e |
| *Heimia salicifolia*,Week 26 | 7.67e |
| *Heimia salicifolia*,Week 30 | 7.33e |
| *Heimia salicifolia*,Week 12 | 6.33e |
| *Heimia salicifolia*,Week 10 | 3.67e |
| *Malus domestica* 'Fuji',Week 18 | 3.67e |
| *Malus domestica* 'Fuji',Week 16 | 3.67e |
| *Heimia salicifolia*,Week 8 | 3.33e |
| *Chaenomeles speciosa*,Week 16 | 3.33e |
| *Malus domestica* 'Fuji',Week 22 | 3.33e |
| *Malus domestica* 'Fuji',Week 26 | 3e |
| *Lagerstroemia* 'Spiced Plum',Week 4 | 2.67e |
| *Chaenomeles speciosa*,Week 22 | 2.67e |
| *Chaenomeles speciosa*,Week 26 | 2.67e |
| *Malus domestica* 'Fuji',Week 20 | 2.67e |
| *Malus domestica* 'Fuji',Week 30 | 2.33e |
| *Chaenomeles speciosa*,Week 20 | 2.33e |
| *Chaenomeles speciosa*,Week 18 | 2.33e |
| *Malus domestica* 'Fuji',Week 14 | 2e |
| *Malus domestica* 'Fuji',Week 12 | 1.67e |
| *Chaenomeles speciosa*,Week 30 | 1.67e |
| *Malus domestica* 'Red Delicious',Week 16 | 1.33e |
| *Malus domestica* 'Fuji',Week 8 | 1e |
| *Malus domestica* 'Fuji',Week 10 | 1e |
| *Chaenomeles speciosa*,Week 12 | 1e |
| *Chaenomeles speciosa*,Week 14 | 0.67e |
| *Chaenomeles speciosa*,Week 10 | 0.67e |
| *Malus domestica* 'Red Delicious',Week 14 | 0.67e |
| *Malus domestica* 'Red Delicious',Week 18 | 0.67e |
| *Lagerstroemia* 'Spiced Plum',Week 3 | 0.33e |
| *Malus domestica* 'Red Delicious',Week 30 | 0.33e |
| *Chaenomeles speciosa*,Week 8 | 0.33e |
| *Malus domestica* 'Red Delicious',Week 12 | 0.33e |
| *Malus domestica* 'Red Delicious',Week 26 | 0.33e |
| *Malus domestica* 'Red Delicious',Week 22 | 0.33e |
| *Malus domestica* 'Red Delicious',Week 20 | 0.33e |
| *Rubus* 'Navaho',Week 30 | 0e |
| *Rubus* 'Navaho',Week 18 | 0e |
| *Rubus idaeus* 'Dorman Red',Week 6 | 0e |
| *Rubus*’Arapaho’,Week 6 | 0e |
| *Rubus fruticosus* ‘Prime Ark Freedom’,Week 6 | 0e |
| *Malus angustifolia*,Week 6 | 0e |
| *Malus domestica* 'Red Delicious',Week 6 | 0e |
| *Rubus*’Arapaho’,Week 3 | 0e |
| *Malus domestica* 'Fuji',Week 6 | 0e |
| *Rubus* 'Navaho',Week 12 | 0e |
| *Rubus*’Arapaho’,Week 4 | 0e |
| *Rubus fruticosus* ‘Prime Ark Freedom’,Week 30 | 0e |
| *Malus angustifolia*,Week 30 | 0e |
| *Heimia salicifolia*,Week 3 | 0e |
| *Heimia salicifolia*,Week 6 | 0e |
| *Diospyros rhombifolia*,Week 3 | 0e |
| *Rubus idaeus* 'Dorman Red',Week 3 | 0e |
| *Rubus idaeus* 'Dorman Red',Week 10 | 0e |
| *Rubus*’Arapaho’,Week 8 | 0e |
| *Rubus*’Arapaho’,Week 10 | 0e |
| *Buxus harlandii*,Week 20 | 0e |
| *Rubus* 'Navaho',Week 26 | 0e |
| *Malus angustifolia*,Week 3 | 0e |
| *Rubus*’Arapaho’,Week 30 | 0e |
| *Buxus microphylla* var. koreana x *Buxus sempervirens* ‘Green Gem’,Week 14 | 0e |
| *Malus angustifolia*,Week 8 | 0e |
| *Chaenomeles speciosa*,Week 3 | 0e |
| *Diospyros virginiana*,Week 3 | 0e |
| *Heimia salicifolia*,Week 4 | 0e |
| *Rubus idaeus* 'Dorman Red',Week 8 | 0e |
| *Rubus idaeus* 'Dorman Red',Week 30 | 0e |
| *Malus domestica* 'Fuji',Week 3 | 0e |
| *Rubus fruticosus* ‘Prime Ark Freedom’,Week 8 | 0e |
| *Diospyros rhombifolia*,Week 14 | 0e |
| *Buxus harlandii*,Week 22 | 0e |
| *Rubus fruticosus* ‘Prime Ark Freedom’,Week 3 | 0e |
| *Rubus idaeus* 'Dorman Red',Week 4 | 0e |
| *Buxus microphylla* var. koreana x *Buxus sempervirens* ‘Green Gem’,Week 20 | 0e |
| *Malus domestica* 'Red Delicious',Week 8 | 0e |
| *Rubus fruticosus* ‘Prime Ark Freedom’,Week 10 | 0e |
| *Diospyros rhombifolia*,Week 6 | 0e |
| *Diospyros rhombifolia*,Week 8 | 0e |
| *Diospyros virginiana*,Week 6 | 0e |
| *Malus angustifolia*,Week 4 | 0e |
| *Buxus microphylla* var. koreana x *Buxus sempervirens* ‘Green Gem’,Week 22 | 0e |
| *Buxus harlandii*,Week 3 | 0e |
| *Buxus microphylla* var. koreana x *Buxus sempervirens* ‘Green Gem’,Week 3 | 0e |
| *Malus domestica* 'Red Delicious',Week 3 | 0e |
| *Rubus fruticosus* ‘Prime Ark Freedom’,Week 4 | 0e |
| *Buxus harlandii*,Week 26 | 0e |
| *Malus angustifolia*,Week 10 | 0e |
| *Diospyros virginiana*,Week 14 | 0e |
| *Diospyros rhombifolia*,Week 4 | 0e |
| *Buxus microphylla* var. koreana x *Buxus sempervirens* ‘Green Gem’,Week 12 | 0e |
| *Buxus harlandii*,Week 12 | 0e |
| *Malus domestica* 'Red Delicious',Week 10 | 0e |
| *Buxus microphylla* var. koreana x *Buxus sempervirens* ‘Green Gem’,Week 26 | 0e |
| *Chaenomeles speciosa*,Week 6 | 0e |
| *Diospyros virginiana*,Week 4 | 0e |
| *Malus domestica* 'Fuji',Week 4 | 0e |
| *Rubus* 'Navaho',Week 8 | 0e |
| *Rubus* 'Navaho',Week 20 | 0e |
| *Buxus harlandii*,Week 14 | 0e |
| *Malus domestica* 'Red Delicious',Week 4 | 0e |
| *Rubus* 'Navaho',Week 16 | 0e |
| *Diospyros rhombifolia*,Week 10 | 0e |
| *Diospyros rhombifolia*,Week 30 | 0e |
| *Rubus* 'Navaho',Week 10 | 0e |
| *Buxus harlandii*,Week 4 | 0e |
| *Buxus harlandii*,Week 18 | 0e |
| *Buxus microphylla* var. koreana x *Buxus sempervirens* ‘Green Gem’,Week 16 | 0e |
| *Buxus microphylla* var. koreana x *Buxus sempervirens* ‘Green Gem’,Week 18 | 0e |
| *Chaenomeles speciosa*,Week 4 | 0e |
| *Rubus* 'Navaho',Week 14 | 0e |
| *Diospyros rhombifolia*,Week 12 | 0e |
| *Diospyros rhombifolia*,Week 26 | 0e |
| *Diospyros rhombifolia*,Week 22 | 0e |
| *Malus angustifolia*,Week 12 | 0e |
| *Rubus*’Arapaho’,Week 14 | 0e |
| *Rubus idaeus* 'Dorman Red',Week 12 | 0e |
| *Rubus idaeus* 'Dorman Red',Week 14 | 0e |
| *Diospyros rhombifolia*,Week 18 | 0e |
| *Diospyros virginiana*,Week 8 | 0e |
| *Malus angustifolia*,Week 14 | 0e |
| *Rubus*’Arapaho’,Week 12 | 0e |
| *Buxus harlandii*,Week 10 | 0e |
| *Buxus microphylla* var. koreana x *Buxus sempervirens* ‘Green Gem’,Week 4 | 0e |
| *Diospyros rhombifolia*,Week 20 | 0e |
| *Buxus microphylla* var. koreana x *Buxus sempervirens* ‘Green Gem’,Week 8 | 0e |
| *Buxus microphylla* var. koreana x *Buxus sempervirens* ‘Green Gem’,Week 10 | 0e |
| *Rubus fruticosus* ‘Prime Ark Freedom’,Week 12 | 0e |
| *Buxus harlandii*,Week 6 | 0e |
| *Buxus harlandii*,Week 8 | 0e |
| *Buxus harlandii*,Week 16 | 0e |
| *Buxus microphylla* var. koreana x *Buxus sempervirens* ‘Green Gem’,Week 6 | 0e |
| *Diospyros virginiana*,Week 10 | 0e |
| *Diospyros rhombifolia*,Week 16 | 0e |
| *Diospyros virginiana*,Week 30 | 0e |
| *Rubus fruticosus* ‘Prime Ark Freedom’,Week 14 | 0e |
| *Diospyros virginiana*,Week 12 | 0e |
| *Malus angustifolia*,Week 26 | 0e |
| *Rubus* 'Navaho',Week 22 | 0e |
| *Diospyros virginiana*,Week 26 | 0e |
| *Diospyros virginiana*,Week 22 | 0e |
| *Rubus fruticosus* ‘Prime Ark Freedom’,Week 18 | 0e |
| *Rubus idaeus* 'Dorman Red',Week 18 | 0e |
| *Rubus idaeus* 'Dorman Red',Week 26 | 0e |
| *Rubus*’Arapaho’,Week 26 | 0e |
| *Diospyros virginiana*,Week 18 | 0e |
| *Diospyros virginiana*,Week 20 | 0e |
| *Rubus fruticosus* ‘Prime Ark Freedom’,Week 26 | 0e |
| *Rubus idaeus* 'Dorman Red',Week 16 | 0e |
| *Malus angustifolia*,Week 18 | 0e |
| *Rubus idaeus* 'Dorman Red',Week 22 | 0e |
| *Rubus fruticosus* ‘Prime Ark Freedom’,Week 22 | 0e |
| *Rubus fruticosus* ‘Prime Ark Freedom’,Week 16 | 0e |
| *Rubus*’Arapaho’,Week 16 | 0e |
| *Malus angustifolia*,Week 22 | 0e |
| *Rubus*’Arapaho’,Week 18 | 0e |
| *Rubus*’Arapaho’,Week 22 | 0e |
| *Diospyros virginiana*,Week 16 | 0e |
| *Rubus idaeus* 'Dorman Red',Week 20 | 0e |
| *Rubus fruticosus* ‘Prime Ark Freedom’,Week 20 | 0e |
| *Rubus* 'Navaho',Week 4 | 0e |
| *Malus angustifolia*,Week 16 | 0e |
| *Rubus* 'Navaho',Week 6 | 0e |
| *Buxus harlandii*,Week 30 | 0e |
| *Buxus microphylla* var. koreana x *Buxus sempervirens* ‘Green Gem’,Week 30 | 0e |
| *Rubus*’Arapaho’,Week 20 | 0e |
| *Malus angustifolia*,Week 20 | 0e |
| *Rubus* 'Navaho',Week 3 | 0e |

^Z^ Means within each column followed by the same letter are not significantly different according to All Pairs, Tukey Honestly Significant Difference at 0.05 confidence level.
